# B cells enhance IL-1 beta driven invasiveness in triple-negative breast cancer

**DOI:** 10.21203/rs.3.rs-5153341/v1

**Published:** 2024-12-31

**Authors:** Nicole J Toney, Lynn M. Opdenaker, Lisa Frerichs, Shirin R. Modarai, Aihui Ma, Holly Archinal, Grace O Ajayi, Jennifer Sims-Mourtada

**Affiliations:** Helen F Graham Cancer Center, and Research Institute; Helen F Graham Cancer Center, and Research Institute; Helen F Graham Cancer Center, and Research Institute; Helen F Graham Cancer Center, and Research Institute; Helen F Graham Cancer Center, and Research Institute; Helen F Graham Cancer Center, and Research Institute; Helen F Graham Cancer Center, and Research Institute; Helen F Graham Cancer Center, and Research Institute

**Keywords:** Breast cancer, Triple negative breast cancer, Interleukin 1 beta, B cells, NFkappa B

## Abstract

Triple-negative breast cancer (TNBC) is an aggressive subtype often characterized by high lymphocyte infiltration, including tumor-infiltrating B cells (TIBs). These cells are present even in early stages of TNBC and associated with microinvasion. This study shows that co-culturing TNBC cells with B cells increases Interleukin-1β (IL-1β) expression and secretion. We further show that B cell-induced IL-1β activates NFκB signaling, leading to higher expression of target genes and promoting IL-1β-dependent increases in matrix metalloproteinase (MMP) activity, invasion, and migration. Immunohistochemical analysis of IL-1β and TIBs in triple-negative ductal carcinoma in situ (DCIS, n=90) and invasive TNBC (n=171) revealed that in DCIS, TIBs correlated with IL-1β expression and microinvasion, with IL-1β also linked to recurrence. In invasive TNBC, IL-1β expression correlated with TIB density and stage, with high IL-1β levels associated with poorer survival outcomes. These findings suggest that early B cell presence in TNBC can induce IL-1β secretion, enhancing invasion and mobility through IL-1β-NFκB signaling. This highlights the potential of IL-1 inhibitors as preventive and therapeutic options for hormone receptor-negative DCIS and TNBC.

## Background

Triple negative breast cancer (TNBC) constitutes approximately 20% of breast cancers and is defined by lack of expression of the estrogen receptor (ER) and progesterone (PR) receptors and lack of overexpression of human epidermal growth factor receptor 2 (HER2) ^1,2^. TNBC are proliferative high-grade tumors that progress quickly from *in situ* to invasive disease ^3^. Compared to other breast cancer subtypes, TNBC have a higher rate of early local and systemic recurrence and shorter breast-cancer specific survival ^4,5^. The factors that contribute to the aggressiveness of TNBC are not well understood.

In recent years, the tumor microenvironment has emerged as a promising therapeutic target for TNBC. Adaptive immune responses have been extensively studied both as a means of tumor surveillance and tumor progression. Large numbers of tumor infiltrating lymphocytes (TILs) have been observed in TNBC^6,7^ compared to other subtypes of breast cancer. Tumor infiltrating B cells (TIB) are present in the microenvironment of TNBC from the early stages of disease development ^8–11^. In DCIS, high densities of TIBs are associated with features of aggressive disease, including hormone receptor negativity, higher grade, larger tumor size, and microinvasion ^8,12–15^. In invasive breast cancers, TIB are associated with higher grade tumors, and lymph node positivity, indicating that B cells play a role in tumor aggressiveness ^16^. While several studies report a role for B cells in progression of TNBC, both in early disease, and lymph-node positive states ^8,17,18^, high densities of infiltrating B cells in primary tumors are associated with increased response to neoadjuvant chemotherapy and better outcomes ^7,19^. Although clonal expansion of B cells is associated with good prognosis ^20^, the spatial location and cytokine profiles may influence the role of TIB ^18,21^.

TIB have been shown to drive chronic inflammation in a murine model of inflammation-associated epithelial cancer, contributing to tumor-promoting processes such as angiogenesis, epithelial cell proliferation, and subsequent recruitment of immune cells ^22^. B lymphocyte-derived factors have also been shown to upregulate the expression of proteins and transcription factors involved in pro-inflammatory signaling pathways such as IκB kinase (IKK) complex proteins and STAT3 in solid tumors ^15,23–26^.

Our group has recently shown that interactions between B cells and TNBC result in the upregulation in gene expression of several inflammatory cytokines by tumor cells leading to a chronic inflammatory environment ^27^. Among these cytokines, interleukin 1-β (IL-1β) is known as a master regulator of inflammation and has been shown to promote angiogenesis, invasion, and mobility in breast cancer ^28–31^. Further, IL1β is a key activator of nuclear factor- κB (NFκB), an important regulatory pathway in TNBC ^32^ that drives proliferation, invasiveness and EMT ^33–36^. In invasive breast cancer, the NFκB signaling axis is significantly associated with large tumor size, high grade tumors, and ER and PR negativity ^37^. In this study, we investigate the role of TIB induced activation of the IL-1β-NFκB signaling axis in TNBC cell lines, and explore the relationship between TIB, IL-1β, and clinical outcomes in hormone receptor negative (HR−) DCIS and invasive breast cancer.

## Results

### B cells upregulate the IL-1β-NfkB signaling axis in TNBC

**Figure 1:**
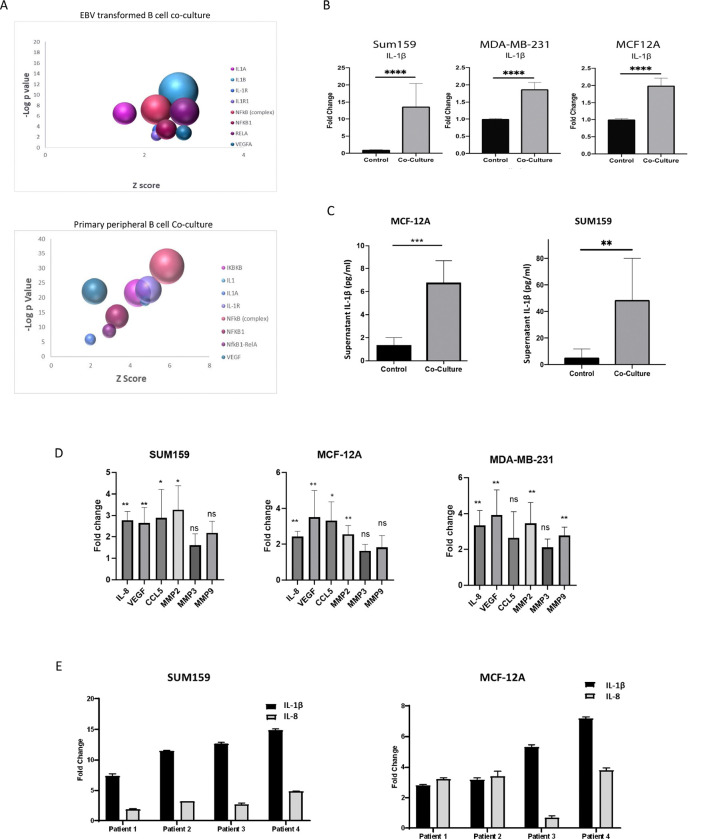
A) Bubble plot of IPA pathway analysis in tumor cells cultured with both EBV transformed B cells or primary peripheral B cells revealed upregulation of regulators related to the NFKB-IL-1 pathway The size of the bubble indicates the number of upregulated genes in the pathway. B) Increases in fold change of IL-1β gene expression of in tumor cells co-cultured for 24 hours with an EBV transformed B cell line as determined by real-time PCR. C) Significant increase in IL-1β protein release from tumor cells is observed by ELISA in MCF-7 and SUM159 cell supernatants 12 hours following 24-hour co-culture with EBV-B cells.D) Significant increases in fold change in gene expression of IL-1β target compared to control cells as determined by real-time PCR in tumor cell lines 24 hours after co-culture with an EBV transformed B cell line. E) Significant increases in fold change of IL-1β and Il-8 gene expression by real-time PCR of in tumor cells co-cultured for 24 hours with primary peripheral B cells from four independent TNBC patients. Graphs show the mean of at least three independent experiments. Error bars represent standard deviations and significance is indicated by asterisk, **** p<.0001,*** p<.001, ** p<.0.05.

To explore the effect of B cells on triple-negative breast cancer (TNBC) cells, we conducted RNA sequencing (RNAseq) on tumor cell lines co-cultured with either primary peripheral B cells from a TNBC patient or an Epstein-Barr virus (EBV)-transformed B cell line. SUM159 and MDA-MB-231 cells were co-cultured with B cells for 24 hours or 4 days, followed by RNAseq analysis. Principal component analysis (PCA) of significantly altered genes showed that gene expression patterns in TNBC cells co-cultured with either EBV-transformed or primary B cells clustered together, suggesting similar effects from both B cell types (**Supplementary Fig. 1a**). Pairwise analysis revealed 1,297 overlapping genes with nominal p < 0.05 and consistent expression direction in both cell lines after 4 days. Notably, IL-1β, a pro-inflammatory cytokine, was among the top upregulated genes in tumor cells co-cultured with both B cell types (**Supplementary Fig. 1b**). Pathway analysis indicated upregulation of the IL-1-NFκB signaling pathway in both co-culture models (Fig. 1a).

Further co-culture studies using three TNBC cell lines (SUM159, MDA-MB-231, and non-invasive MCF12A) confirmed significant increases in IL-1β gene expression in tumor cells when co-cultured with the EBV-transformed B cells (Fig. 1b). Next, we aimed to determine whether the observed increase in IL-1β gene expression translated into protein expression and secretion by the tumor cells. The release of IL-1β is contingent upon the cleavage of its inactive precursor by caspase 1, a key component of the NLRP3 inflammasome complex^38^. To assess whether TNBC cell lines could express activated IL-1β, we performed western blot analysis to detect cleaved caspase 1 in tumor cell lines both before and after co-culture with EBV-transformed B cells. Cleaved caspase 1 was detected in all cell lines, and while there was a slight increase in its expression following co-culture in the MDA-MB-231 and MCF-12A cells, this increase was not statistically significant (**Supplemental Fig. 2**).

We further examined release of IL-1β by an invasive cell line (SUM159) and a non-invasive cell line (MCF-12A) after co-culture by ELISA. To ensure that the IL-1β detected was secreted solely by the tumor cells, we cultured the cell lines with B cells for 24 hours, after which the B cells were removed. The tumor cells were then provided with fresh media and cultured for an additional 12 hours. Supernatants were collected and analyzed using an IL-1β-specific ELISA. This analysis revealed significant increases in IL-1β protein levels in both cell lines after co-culture (Fig. 1c). Additionally, we observed upregulation of IL-1β target genes IL-8, VEGF, CCL5, and MMP2 in SUM159, MCF12A and MDA-MB-231 cells (Fig. 1d) This upregulation indicates functional activation of IL-1β signaling in tumor cells co-cultured with B cells. To ensure that the observed increase in IL-1β was not an artifact of the EBV-transformed B cell line, we conducted co-culture experiments using primary peripheral B cells isolated from four different TNBC patients. B cell purity was confirmed by flow cytometry (**Supplemental Fig. 3**). As shown in Fig. 1e, significant upregulation of IL-1β was observed in both SUM159 and MCF12A cells after co-culture with primary B cells. Moreover, we also observed significant upregulation of IL-8 gene expression, a known target of IL-1β signaling. These findings are consistent with the RNAseq data obtained from both peripheral and EBV-transformed B cells, demonstrating that both B cell models similarly induce IL-1β upregulation and activate downstream signaling pathways.

**Figure 2:**
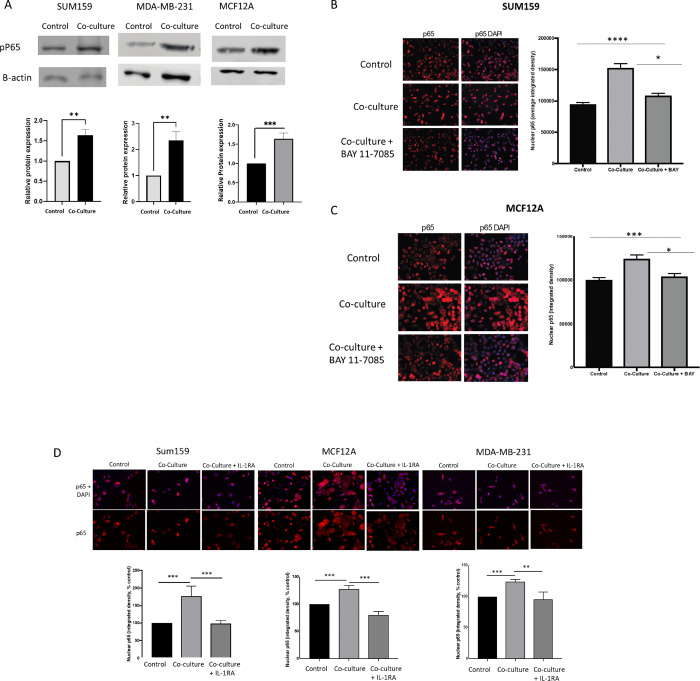
A) Western blot analysis showing significantly increased phosphorylation of the p65 subunit of the NFκB complex in tumor cells 24 hours after co-culture with EBV-B cells as compared to control cells. B) Nuclear localization of P65 after co-culture with EBV-B cells can be blocked by addition of IKK inhibitor Bay 11–7085 in SUM159 (B) and MCF12A (C) cells. D) Nuclear localization of P65 after co-culture with EBV-B cells can beblocked by addition of an IL-1 receptor antagonist (IL-1RA) in TNBC cells. Graphs show the mean of atleast three independent experiments. Error bars represent standard deviations and significance is indicated by asterisk, *** p<.001, ** p<.0.01.

To further confirm IL-1β signaling, we tested the ability of B cells to activate IL-1β-dependent NFκB signaling in tumor cells by assessing phosphorylation of serine 536 of the p65 (RELA) subunit. Phosphorylation of p65 at this site by IL-1 induced IKK activity is essential for nuclear localization and transcriptional activity of the NFκB signaling complex^39^. As shown in Fig. 2a, we observed an increase in the expression of the phosphorylated form of p65 (phospho-p65) by western blot in all three TNBC cell lines after co-culture with B cells, indicating activation of the NFκB signaling pathway. Additionally, immunostaining experiments demonstrated that p65 nuclear localization was elevated in SUM159 cells as early as 1 hour after co-culture with B cells, with further increases observed up to 24 hours (Fig. 2b, **Supplementary Fig. 4**). This nuclear translocation of p65 was effectively blocked by BAY 11–705, an inhibitor of IKK, in both SUM159 and MCF12A cells, confirming the activation of canonical NFκB signaling (Figs. 2c & 2d).

To determine whether B cell-induced NFκB activation was mediated by increased IL-1β expression, we repeated the p65 nuclear localization studies with the addition of AF12198, a small molecule inhibitor of the IL-1 receptor (interleukin 1 receptor antagonist, IL-1RA)^40^. Addition of IL-1RA for 24 hours during B cell co-culture restored nuclear localization of p65 to control levels in all three cell lines (Fig. 2e), indicating B cell induced NFκB occurs specifically through IL-1 signaling.

### B cells increase TNBC invasiveness and migration through an IL-1β - dependent mechanism

IL-1β signaling has been shown to induce invasiveness and migration of TNBC ^28,29^. Pathway analysis of RNAseq data from TNBC cell lines co-cultured with B cells revealed significant upregulation of pathways associated with tumor cell migration, invasion, and cell movement **(Supplementary Fig. 5)**. Based on these findings, we investigated whether B cells influenced epithelial-mesenchymal transition (EMT) gene expression, tumor mobility, and invasiveness during co-culture. In SUM159 cells, we observed a significant increase in the expression of the mesenchymal marker vimentin, while MDA-MB-231 cells showed increased expression of N-cadherin accompanied by a corresponding decrease in the epithelial marker E-cadherin upon co-culture with B cells (**Supplementary Fig. 6a&b**). However, these changes in gene expression did not translate to significant alterations at the protein level, likely due to the highly mesenchymal nature of both cell lines, which exhibit high constitutive expression of vimentin (**Supplementary Fig. 6c**). SUM159 cells also show high N-cadherin expression, and both cell lines lack E-cadherin protein expression.

**Figure 3:**
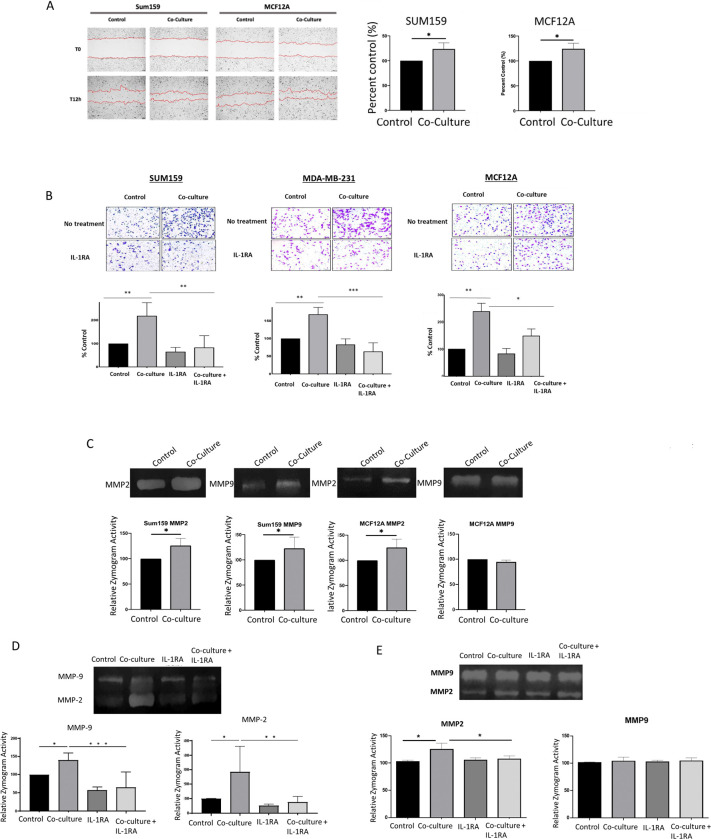
A) Wound healing assay of SUM159 and MCF12A cells following 72-hour co-culture with B lymphocytes. Results were calculated as mean +/− standard deviation of the distance between the leading edges of the wound at 0 and at 12hrs.B) In vitro Matrigel invasion assay using TNBC cell lines cultured cells with and without B cells and IL-1RA treatment. Representative 10x images of randomly selected fields show invaded cells stained with Crystal Violet. C) Zymography analysis of MMP2 and MMP9 in SUM159 and MCF12A cell supernatants following 72-hour co-culture with EBV B cells. Upregulation of MMP activity in zymogram assays is blocked in SUM159 (D) and MCF12A (E) cells co-cultured for 72 hours with EBV B cells in cells co-treated with IL-1RA.Graphs show the mean of at least three independent experiments. Error bars represent standard deviations Significance is indicated by asterisk, *** p<.001, ** p<.0.01, *p<.0.05.

Co-culture resulted in increased migratory activity as measured by distance of wound closure over a 12-hour period in both SUM159 and MCF12A cells. Furthermore, co-culture with B cells resulted in significantly increased invasion of SUM159 and MDA-MB-231 cells through a Matrigel insert, an effect that was reversed by the addition of IL-1RA (Fig. 3a). As expected, MCF12A cells, which are non-invasive, exhibited low invasive capacity compared to the invasive TNBC cell lines. However, upon co-culture with B cells, MCF12A cells demonstrated a significant increase in invasion which was dependent on IL-1β signaling (Fig. 3b.

**Figure 4:**
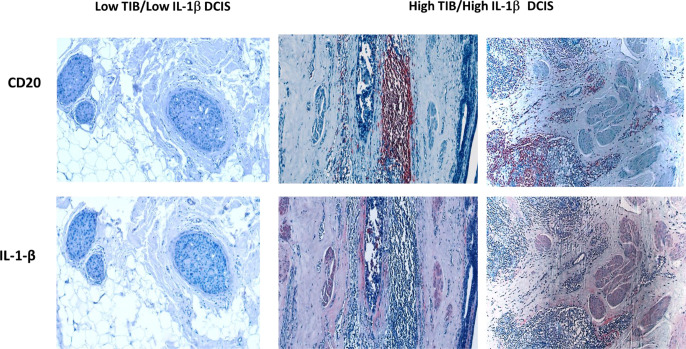
CD20 And IL-1β expression in TN DCIS tissue sections. Immunohistochemical analysis of serial DCIS tissues from two patients showing CD20 (top, red) and IL-1β staining (bottom, red). Sections were counterstained with hematoxylin (blue).

Zymogram analysis revealed increased activity of both MMP-2 and MMP-9 in SUM159 cells following co-culture with B cells. In contrast, MCF12A cells co-cultured with B cells showed an induction of MMP-2 activity only (Fig. 3c). These findings align with the gene expression data presented in Fig. 1c, where MCF12A cells exhibited a significant increase in MMP2 but not MMP9 gene expression. The addition of IL-1RA during co-culture significantly inhibited the activation of both MMP-2 and MMP-9 in SUM159 cells (Fig. 3d) and suppressed MMP-2 activity in MCF12A cells (Fig. 4c) These results underscore the role of IL-1β signaling in promoting tumor cell invasiveness and MMP activation in TNBC.

### Tumor infiltrating B cells correlate with IL-1β and microinvasion in TN DCIS.

To further explore the association between TIB, IL-1β and breast cancer invasiveness, we examined the relationship between TIB density, IL-1β expression, and invasion in tumor microarrays containing tissue from 90 hormone receptor negative DCIS. Patient characteristics are listed in **Table 1**. Serial sections were stained with antibodies against pan B cell marker CD20, and human IL-1β (Fig. 4). Negative control slides were stained using an isotype-matched control antibody (**Supplemental Fig. 7)**.

A TIB score was determined by counting the number of CD20 + B cells per mm^2^ of tumor tissue. Tissues with CD20 density in the top quartile were considered high TIB, while those below the top quartile were considered moderate to low. High TIB infiltration was observed in 29% (26 out of 90) of the samples. High IL-1β expression was defined as staining intensity per area in tumor cells above the median score, observed in 26% (33 out of 90) of samples. A moderate correlation was found between high TIB density and high IL-1β expression (Spearman’s rho = 0.530, p = 0.001; Table 2).

Both high TIB scores and high IL-1β expression were significantly associated with microinvasion (Spearman’s rho = 0.243, p = 0.022; Spearman’s rho = 0.333, p = 0.001, respectively). Notably, only high IL-1β expression was associated with recurrence (Spearman’s rho = 0.275, p = 0.011). These findings suggest that elevated levels of TIBs and IL-1β contribute to the invasiveness of breast cancer, with IL-1β also playing a critical role in tumor recurrence.

### IL-1β expression predicts poor survival in invasive TNBC.

**Figure 5:**
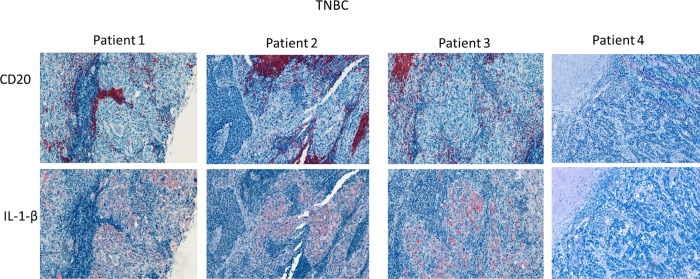
CD20 And IL-1β expression in TNBC. Immunohistochemical analysis of serial sections of tumor from four TNBC patients showing CD20 (top, red) and IL-1β staining (bottom, red). Sections were counterstained with hematoxylin (blue).

We next examined the relationship between TIB density and IL-1β expression in invasive triple-negative breast cancer (TNBC) using tissue microarrays from 171 patients. These samples were stained and scored as described previously (**Table 3**, Fig. 5). Among these samples, 85 (49.7%) exhibited high TIB density, and 54 (31.5%) showed high IL-1β expression. A weak but significant correlation was observed between TIB density and tumor IL-1β expression in invasive TNBC (Spearman’s rho = 0.168, p = 0.024, Table 4). TIB density was not associated with any other patient variables except age, where a weak inverse association was found (Spearman’s rho = −0.176, p = 0.017).

**Figure 6:**
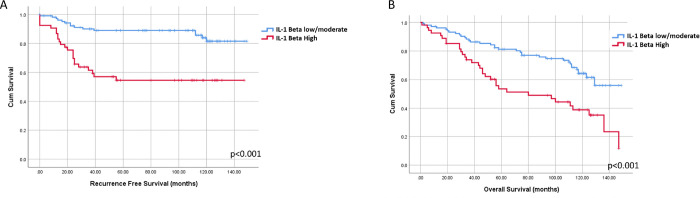
High IL-1β expression but not TIB is correlated with poor outcomes in TNBC. Kaplan-Meyer survival analysis showing (A) shorter recurrence free survival (RFS, p<0.001) and (B) overall survival (OS, p<0.001) in patients with high tumor IL-1β expression.

High tumor IL-1β expression was significantly associated with advanced tumor stage (Spearman’s rho = 0.226, p = 0.001). Kaplan-Meier analysis demonstrated that high IL-1β expression was significantly correlated with poorer recurrence-free and overall survival (Figs. 6a & 6b). In contrast, no significant association was found between TIB density and patient outcomes in this cohort (Supplementary Fig. 8a & 8b). These findings suggest that while TIB density and IL-1β expression are correlated in invasive TNBC, IL-1β plays a more critical role in tumor progression and patient prognosis.

## Discussion

**Figure 7:**
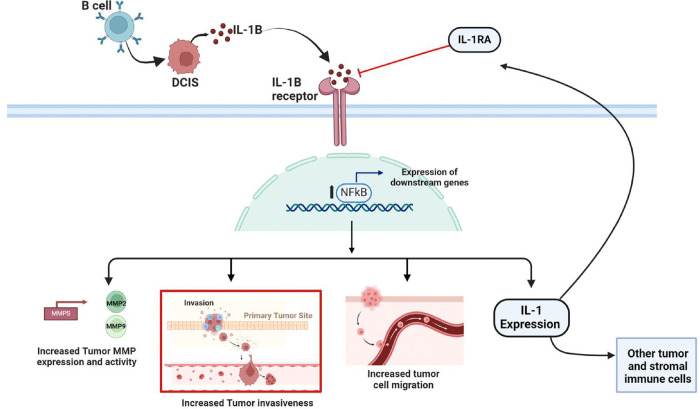
This schematic represents our conclusion, based on our findings, that B cells may promote the aggressive nature of TNBC early in the disease by upregulating activity of IL-1β-NFκB signaling in tumor cells leading to increased motility and invasiveness.

Our findings suggest that B cell infiltration in TNBC occurs early in the disease’s development and may play a crucial role in promoting the invasion and migration of tumor cells, contributing to the progression from in situ to invasive disease. These results align with previous studies that have shown a connection between tumor-infiltrating B cells (TIBs) and microinvasion in DCIS^8,12,14,15^. Our study demonstrates that TIBs induce an inflammatory response leading to the secretion of IL-1β by tumor cells. This secretion, in turn, enhances tumor cell motility and invasiveness through the activation of the IL-1β-NFκB signaling pathway (Fig. 7). Additionally, we show that high tumor expression of IL-1β is associated with poorer outcomes in both DCIS and invasive TNBC, underscoring the critical role of this inflammatory axis in disease progression and prognosis.

Previous studies have established that IL-1β expression in breast tumors of all subtypes is associated with increased migration and metastatic potential ^28,29,41^. Tulotta et al. demonstrated that production of IL-1β by breast tissue in a humanized mouse model enhanced EMT, migration, invasion, and metastasis, and inhibition of IL-1β decreased metastatic potential^28,29^. Other studies have also reported that IL-1β induces NFκB signaling and cell migration^42^,as well as invasiveness ^30^ in TNBC. These findings are consistent with our results, showing that IL-1β-driven NFκB activation, induced by tumor-infiltrating B cells (TIBs), enhances tumor cell migration and invasiveness.

Although our data suggest that tumor-infiltrating B cells (TIBs) promote the aggressiveness of TNBC, it is important to acknowledge that several studies have also highlighted a role for TIBs in anti-tumor immune responses. In particular, TIBs have been shown to support both antibody-driven and cytotoxic T cell responses in TNBC and other cancers^20,43,44^. These anti-tumor activities include the production of antibodies that can target tumor antigens and the promotion of T cell-mediated cytotoxicity, which can help suppress tumor growth and progression ^20,43,44^.

Inflammation induced by TIBs, especially plasmablasts, has been shown to recruit CD8 + T cells into the tumor microenvironment via the expression of T cell–recruiting chemokines, thereby enhancing T cell-driven anti-tumor immunity.^45^ This dual role of TIBs, contributing to both tumor promotion and suppression, underscores the complexity of the tumor microenvironment and suggests that the effects of TIBs on tumor behavior may depend on the specific context and interactions within the tumor milieu.

Our work, along with those of others, demonstrates that the presence of TIBs in the tumor microenvironment contributes to chronic inflammation, partly through the increased expression of inflammatory cytokines by both B cells and tumor cells^27,45^. However, our study did not examine the effects of tumor-secreted IL-1β on TIBs or other immune cells. Nevertheless, TIB-driven increases in IL-1β may have a paradoxical inhibitory effect on tumor immunity. IL-1β is known to promote macrophage M2 polarization, which is associated with a tumor-supportive environment, as well as to impede CD8 + T cell activation and foster resistance to checkpoint inhibitors^46^. Furthermore, IL-1β-NFκB signaling triggers the expression of additional pro-inflammatory cytokines and chemokines that sustain chronic inflammation and angiogenesis within the tumormicroenvironment^47,48^. These findings suggest that the effects of TIBs on tumor progression are complex and may vary depending on tumor-specific characteristics, the presence of particular B cell subsets, or the broader context of the tumor microenvironment.

Our findings of a moderate to strong correlation between TIBs and IL-1β in DCIS samples suggest that B cells may negatively impact early disease stages by activating the IL-1-NFκB signaling pathway and its downstream targets, which promote extracellular matrix degradation and increase invasiveness. This relationship between TIBs and IL-1β expression persisted in invasive cancer, though the association was weaker than in non-invasive disease. Interestingly, IL-1β signaling, rather than TIB presence, was associated with advanced-stage cancer. We hypothesize that TIB may prime TNBC at initial stages to activate signaling pathways such as IL-1β-NFκB that promote advanced diseases. It is also important to note that our study utilized a pan-B cell marker and did not differentiate between B cell subtypes or specific signatures of TIBs that may be linked to poorer outcomes^49^. Further research is needed to identify the specific B cell subtypes that activate IL-1-NFκB signaling in tumor cells and to determine their precise role in TNBC progression. Understanding these dynamics could provide new insights into the development of targeted therapies aimed at modulating the immune environment in TNBC.

Further study is needed to understand the mechanism driving IL-1β expression in tumor cells. Activated B cells are known to secrete inflammatory cytokines such as IL-6, Il-1α, TNF-α and Il-1β itself^45,50^, which may stimulate IL-β secretion and NfκB activation in tumor cells. Furthermore, while it is possible that B-cell secreted IL-1β or other NfκB activating cytokines may have played a role in initial activation of NfκB, secretion of IL-1β and downstream effects were observed even upon removal of B cells. IL-1β is a proinflammatory cytokine and is both an activator and a target of NFκB signaling ^51^ Although IL-1β and TIBs are only weakly correlated in invasive breast cancer, it is possible that the initial activation of IL-1β in a small population of tumor cells by B cells may have triggered a positive feedback loop. This loop could lead to the sustained release of IL-1β from tumor cells and the subsequent induction of IL-1β expression in other tumor cells not directly exposed to B cells. These findings suggest that IL-1β signaling may propagate within the tumor environment independently of B cells, amplifying inflammatory responses and promoting tumor progression through complex interactions involving multiple cell types. While our data highlight the role of IL-1β induction in tumor cells, we cannot entirely rule out the potential contributions of other immune or stromal cells within the tumor microenvironment to tumor IL-1β expression. Further research is needed to fully elucidate the interplay between different cell populations in the tumor microenvironment and their collective impact on IL-1β signaling and cancer progression.

In breast cancer, activated NFκB is detected predominantly in hormone receptor negative tumors ^52^. Previous studies suggest that NFκB activation may be important in the formation of TNBC. Aberrant activation of NFκB in the normal mouse mammary epithelium after development results in hyperproliferative, enlarged ducts with filled lumens resembling DCIS ^53^. In this model, elevated levels of inflammatory markers expressed by epithelial cells correlated with down-regulation of hormone receptors and markers of epithelial differentiation. Consistent with these findings, our study demonstrated that B cells could induce invasion and MMP expression in the non-invasive MCF-12A cell line. This supports the hypothesis that inflammation and early NFκB activation in abnormal breast cell growth may contribute to the formation of triple-negative DCIS and its progression to invasive cancer. The ability of B cells to drive these processes in non-invasive cells highlights the potential of B cell-induced IL1β-NFκB signaling to act as early drivers of malignancy, particularly in the context of hormone receptor-negative breast cancers.

## Conclusions

In summary, our findings indicate that B cells may promote the aggressive nature of TNBC early in the disease by upregulating activity of IL-1β-NFκB signaling in tumor cells leading to increased motility and invasiveness. We show that the presence of TIBs in triple-negative DCIS is associated with IL-1β expression and microinvasion, while recurrence is linked specifically to IL-1β. The association between TIBs and IL-1β persists in invasive breast cancer, with strong tumor IL-1β expression correlating with poor outcomes in invasive disease.

We propose that high densities of TIBs may promote aggressive tumor characteristics by activating the IL-1β-NFκB pathway early in the disease, a role that may be distinct from their involvement in anti-tumor immune responses. These findings align with other studies suggesting that IL-1β signaling could be a valuable pharmacologic target for improving outcomes in TNBC.

## Materials and Methods

### Cell culture and inhibitors

MDA-MB-231 and MCF12A cells were obtained from American Type Culture Collection (ATCC; Virginia, USA). SUM159 cells were obtained from Asterand Minnesota, USSI). MDA-MB-231 cells were maintained in RPMI-1640 Medium (ThermoFisher Scientific, Massachusetts, USA) + 10% FBS and 1x antibiotic/antimycotic (ThermoFisher Scientific). SUM159 cells were cultured in Ham’s F12 medium (ThermoFisher Scientific) + 10% FBS, 1x antibiotic/antimycotic, 2 μg/ml insulin (ThermoFisher Scientific), and 0.1mg/ml hydrocortisone (Sigma Aldrich). MCF12A cells were cultured in a 1:1 mixture of Dulbecco’s modified Eagle’s medium and Ham’s F12 medium (Life Technologies) supplemented with 5% horse serum (ThermoFisher Scientific), 0.01 mg/ml human insulin, 100 ng/ml cholera toxin (Sigma Aldrich), 20 ng/ml human epidermal growth factor (ThermoFisher Scientific), 500 ng/ml hydrocortisone (Sigma Aldrich), and 1x antimycotic/antibiotic TNBC patient blood was received under an institutional review board (IRB)-approved protocol and PBMCs were obtained using Ficoll-Paque plus (Millipore Sigma, Rockeville MD) and then B cells were isolated via magnetic bead sorting using the B lymphocyte Isolation Kit II, human (Miltenyi Biotech; Bergisch Gladbach, Germany) according to manufacturer’s instructions. EVB-transformed and primary B cells were cultured in RPMI plus 10% FBS overnight and then plated for co-culture.

For co-culutre studies TNBC cells were seeded on the lower chamber of a 6-well plate in TNBC cell growth media and B cells in RPMI media were added to the upper chamber separated by a 0.4 μm filter (Thermo Fisher Scientific). Controls contain the same media ratio without the B cells. Bay-11–705, an inhibitor to IKKB, was purchased from Tocris, and used at a concentration of 5μM. AF12198 (refer to as IL-1RA in text) (Bio-Techne Corporation; Minnesota, USA) was added to cultures at a concentration of 1 μM.

### RNA Sequencing

Tumor cells were co-cultured for 24 hours, or 4 days with primary or EBV-transformed B cell line, and total RNA was extracted using Trizol following manufacturer’s instructions Libraries were prepared using ScriptSeq RNA library prepV2 (Illumina), and sequencing was performed on Illumina Nextseq500 on the high-output mode in a 75 bp single-end run. RNA-seq data were aligned using RSEM along with Bowtie2. Paired analysis was performed to combine SUM159 and MDA-231 replicates resulting in a single overlapping gene set for analysis (1297 genes for 24 hours, 1036 genes for 4-day analysis). All reads within any transcript coding part of a gene were counted to get expression for each gene. Raw counts were tested for differential expression using DESeq2 method. Deseq2 normalized count values were used for heatmap visualization of expression differences in genes with at least 10 raw counts in at least one sample. Edge R was used to identify differential expression changes among groups. IPA analysis was performed to determine affected pathways and gene regulators. Principle component analysis was done in cell lines with and without co-culture with EBV-transformed B cells or primary B cells using genes that showed significant changes in gene expression upon co-culture with EVB cells.

### Immunofluorescence

Tumor cells were plated on glass coverslips and co-cultured with B cells as described above. Cells were fixed with methanol, blocked and incubated overnight with an antibody to p65 (#D14E12, monoclonal rabbit anti-human, 1:400; Cell Signaling Technology, Massachusetts, USA), washed and then incubated with a poly clonal donkey anti-rabbit antibody (Alexa Flour 555, #A31572, Thermo Fisher Scientific), washed and mounted on glass slides using Prolong^®^ Gold Antifade Mountant with DAPI (Thermo Fisher Scientific). Images were captured using a ZEISS Observer Z1 microscope with an AxioCam MRm camera. Four 20x fields of view were captured, and the integrated density was calculated for each cell in all four fields of view. The mean gray values of all cells in all four fields of view were averaged to determine the average amount of nuclear p65 in each treatment condition. Experiments were repeated in triplicate.

### Real Time Quantitative PCR

Following co-culture and inhibitor treatment, RNA was extracted using TRIzol^®^ Reagent per the manufacturer’s instructions and cDNA conversion and real time PCR were performed as previously described ^27^. Primer sequences are listed in supplemental table 1. Experiments were performed in triplicate.

### Western Blotting

Cell lysates were obtained in RIPA buffer with phosphatase and protease inhibitors and western blotting was performed as previously described^54^. Primary antibodies and secondary antibodies are listed in supplementary table 2. Blots were imaged and quantified on the Li-Cor Odyssey FC. Experiments were performed in triplicate.

### Enzyme-Linked Immunosorbent Assay

Following 24-hour co-culture, B cells and media were removed, and 1.5 ml of fresh media was added back to wells for a 12-hour period. Supernatants were then collected, spun down to remove cell debris, and frozen at −80°C until further use. Samples were analyzed using the Human IL-1 beta/IL-1F2 Quantikine ELISA Kit (Bio-Techne Corporation), according to manufacturer’s instructions, using equal volumes of control and treated samples. Samples were read at 450 nm absorbance on the Infinite^®^ 200 PRO NanoQuant (Tecan Life Sciences). Samples were prepared in duplicate and each ELISA was repeated at least twice.

### Invasion Assays

IL-1RA treatment was administered co-culture and invasion assays as described above Following co-culture, B cells were removed, and cancer cells were counted. Corning^®^ BiocoatTM Cell Culture Inserts were rehydrated as described by the manufacturer. Cell lines were added to the upper chamber in 0.5 ml serum-free media. Complete cell culture media was used as a chemoattractant. Cells invaded for 24–48 hours, with or without presence of B cells, and with or without 1 μM AF12198 in the upper chamber, washed, and gels were removed. Cells adhered to the bottom of the membrane were fixed in 100% methanol cooled at −20°C for 5 minutes, and then stained in Crystal Violet for 5 minutes, rinsed and dried. Ten 10x fields of view were obtained from each membrane and counted. The total count for all fields was tabulated per sample and the percentage compared to control samples was calculated. Experiments were performed in triplicate for SUM159 and MDA-MB-231. Experiments were repeated four times for MCF-12A.

### Zymography

Following 24-hour co-culture, B cells were removed, tumor cells were washed and cultured an addition 12 hours and tumor cell media was collected, loaded onto a 7.5% poly-acrylamide gelatin-embedded gel, renatured, and then incubated for 16 hours at 37°C. The gels were stained with Coomassie and then de-stained for 5 minutes. Gels were imaged on the Li-Cor Odyssey FC. Bands were quantified in ImageJ. Experiments were performed in triplicate.

### Wound Healing Assays

Following 72-hour co-culture, B cells were removed, and tumor cells were scratched, washed then fresh media was added back to cells and images were taken at time 0 and 12 hours after incubation. Images were quantified by taking ten measurements of distance between leading edges of the scratch in each image and the average change in distance was calculated Experiments were performed in triplicate.

### Immunohistochemical procedure

Paraffin-embedded tissue microarrays containing 5mm cores of tissue were obtained under an IRB approved protocol and analyzed by immunohistochemistry with the following primary antibodies: IL-1β (#sc-32294, monoclonal mouse anti-human, 1:50, Santa Cruz Biotechnology41,42), or CD20 (#ab9475, monoclonal mouse anti-human, 1:100, Abcam). Negative control slides were performed as previously described with omission of the primary antibody ^27^.

Slides were scored by at least two researchers blinded to the sample data. CD20 was scored by overlaying a 20×20 grid of 1 mm squares. The density score was used to discriminate high vs low/moderate density B cells, with the top quartile being considered high density. For IL-1β staining, images were captured using a Zeiss Axio microscope using a 10X objective. Segmentation of tumor cells and measurement of the sum staining intensity per area was performed using Zen Blue.

### Statistical analysis

Statistical tests were performed in Graphpad Prism 8 and SPSS (IBM). PCA analysis was performed in Graphpad prism 9. For PCA analysis the top 100 significant differentially expressed genes were used for comparison.

## Data Availability

Sequence data contains patient samples and will be provided on an individual basis per request.
